# Effectiveness of a Surgery Admission Unit for patients undergoing major elective surgery in a tertiary university hospital

**DOI:** 10.1186/1472-6963-10-23

**Published:** 2010-01-22

**Authors:** B Ortiga, C Capdevila, A Salazar, MF Viso, C Bartolomé, X Corbella

**Affiliations:** 1 Medical Coordinator. Hospital Universitari de Bellvitge. IDIBELL. Universitat de Barcelona. Feixa Llarga, s/n.08907. L'Hospitalet de Llobregat, Barcelona. Spain; 2 Medical Subdirector. Hospital Universitari de Bellvitge. IDIBELL. Universitat de Barcelona. Feixa Llarga, s/n.08907. L'Hospitalet de Llobregat, Barcelona. Spain; 3 Medical Director. Hospital Universitari de Bellvitge. IDIBELL. Universitat de Barcelona. Feixa Llarga, s/n.08907. L'Hospitalet de Llobregat, Barcelona. Spain; 4 Nursing Coordinator. Hospital Universitari de Bellvitge. IDIBELL. Universitat de Barcelona. Feixa Llarga, s/n.08907. L'Hospitalet de Llobregat, Barcelona. Spain; 5 Chief Executive. Hospital Universitari de Bellvitge. IDIBELL. Universitat de Barcelona. Feixa Llarga, s/n.08907. L'Hospitalet de Llobregat, Barcelona. Spain

## Abstract

**Background:**

The increasing demand on hospitalisation, either due to elective activity from the waiting lists or due to emergency admissions coming from the Emergency Department (ED), requires looking for strategies that lead to effective bed management. The aim of this study was to evaluate the effectiveness of a surgery admission unit for major elective surgery patients who were admitted for same-day surgery.

**Methods:**

We included all patients admitted for elective surgery in a university tertiary hospital between the 1st of September and the 31st of December 2006, as well as those admitted during the same period of 2008, after the introduction of the Surgery Admission Unit. The main outcome parameters were global length of stay, pre-surgery length of stay, proportion of patients admitted the same day of the surgery and number of cancellations. Differences between the two periods were evaluated by the T-test and Chi-square test. Significance at P < 0.05 was assumed throughout.

**Results:**

We included 6,053 patients, 3,003 during 2006 and 3,050 patients during 2008. Global length of stay was 6.2 days (IC 95%:6.4-6) in 2006 and 5.5 days (IC 95%:5.8-5.2) in 2008 (p < 0.005). Pre-surgery length of stay was reduced from 0.46 days (IC 95%:0.44-0.48) in 2006 to 0.29 days (IC 95%:0.27-0.31) in 2008 (p < 0.005). The proportion of patients admitted for same-day surgery was 67% (IC 95%:69%-65%) in 2006 and 76% (IC 95%:78%-74%) in 2008 (p < 0.005). The number of cancelled interventions due to insufficient preparation was 31 patients in 2006 and 7 patients in 2008.

**Conclusions:**

The implementation of a Surgery Admission Unit for patients undergoing major elective surgery has proved to be an effective strategy for improving bed management. It has enabled an improvement in the proportion of patients admitted on the same day as surgery and a shorter length of stay.

## Background

Admission to an acute hospital as an inpatient is a major event for people, either coming as an elective admission or coming from an Emergency Department (ED). However, hospitals have a fixed capacity in order to meet the demand of both areas. In some periods of the year, general public hospitals suffer from peaks in emergency admissions, basically due to respiratory and cardiovascular related diseases [1,2], which influences the rate of elective admissions and hence waiting times for admission. It has been proved that there are complex relationships between such factors and clinical as well as managerial decision making [3]. One of the most visible organizational changes is a shorter hospital stay, especially for elective surgery [4], which includes increased use of day surgery, improved discharge planning and post-acute care at home, less invasive surgical techniques and improved anaesthetic drugs [5].

The provision of inpatient beds is central to the acute hospital service: patients do not have to wait when they need a bed in an ED; planned admissions for surgery are not cancelled because of a lack of beds; and patients are admitted to wards that are appropriate to their clinical needs.

Several studies have demonstrated that there is not any clinical reason for not admitting on the same day as surgery in elective surgery, except in a few cases [6-8]. Traditionally, all surgical patients are hospitalised at least one day prior to surgery for being clerked, investigated and evaluated by the anaesthesiologist [9]. At the moment, the preoperative anaesthetic assessment is performed in an outpatient visit and usually when the patient that is admitted to the hospital is waiting for a surgical procedure. Only a few cases require specific preoperative arrangements for surgery that recommend an admission the day before the intervention. However, there still are admissions the day before in order to avoid cancellations due to the lack of ward beds or to avoid delays in going to the theatre for earlier procedures [10].

The aim of this study was to evaluate the effectiveness of a Surgery Admission Unit in the reception of patients undergoing major elective surgery.

## Methods

This study was set in a 900-bed university tertiary hospital located in the metropolitan area of Barcelona (Spain). It belongs to the Spanish National Health Service, attends more than 120,000 emergency visits annually and the mean number of monthly elective admissions is 1,650 (IC 95%: 1.609-1.691), without day surgery. For our study, we included patients who underwent elective major surgery between the 1^st ^of September and the 31^st ^of December 2006 as well as those admitted during the same period of 2008, after the introduction of the Surgery Admission Unit.

The unit was comprised of 10 points of attendance located in three hospital wards. They were basically chairs for treatment and they were coordinated and organized by nurses who performed the reception of the patient and the preoperative preparation. Chair assignment was performed by the admissions team, who was also in charge of the hospital's bed management. Each chair allowed a maximum of three preparations per day, so the maximum capacity was 30 patients each day. In general, patients were admitted to the hospital two hours before surgery. Special instructions, diagnostic techniques, investigations or treatments from the admitting doctor were written in the admission form, as well as the inappropriateness of the preparation in a chair due to mobilization difficulties or any other reason. Patients were admitted to these chairs from 7:00 am to 12:00 am. There was a double nursing staff between 7:00 am to 8:00 am, as nurses begun one hour earlier while the night staff was still in the ward. That was the time when there was the highest number of patients to prepare. The expected maximum length of stay in the Admissions Unit ranged from 2-3 hours. Day surgery, minor surgery and emergency surgery were excluded from the study. General preparation pathways were uploaded to the hospital intranet.

Bed management was done through a centralized team, who placed emergency and elective patients in the most appropriate beds, allowed patient transfers between wards and checked patient discharge, in order to have a correct patient allocation and a global vision of the occupancy at any moment [11]. In this context, chairs were used to prepare patients whose expected length of stay was over 48 hours, and during the surgery and recovery process there was time to get available beds as a consequence of the discharges of the day. Patients who underwent day surgery were admitted to a day surgery hospital and patients who were undergoing a short length of stay surgery also had a special ward that closed at weekends.

The following variables were recorded: patient demographics, main diagnosis and procedure, length of stay, causes of delay in sending the patient to theatre and reasons for cancellation. We did not look for ethical approval as the organizational change described in this study did not cause any change in the clinical management of the patients.

The main outcome measures were frequency of incidences that affected the theatre, surgery cancellations due to lack of preparation, proportion of patients admitted the same day as surgery, global length of stay, pre-surgery length of stay, mean number of emergency patients without a bed at 8:00 am and the percentage of emergency patients admitted.

Descriptive statistics in the form of percentages and means as well as their confidence intervals are presented. All statistical analysis was conducted using the Statistical Software Program (SPSS, Chicago IL) for Windows (version 14) [12]. Differences between the two periods were evaluated by the T-test and Chi-square test. Significance at P < 0.05 was assumed throughout.

## Results

We included 6,053 major surgery interventions, of which 3,003 were done in 2006 and 3,050 were done during the same period of 2008. In Table 1 all the hospital's surgery specialities are listed with the number of all the admissions during the study period in 2006 and 2008. The number of these admissions that were for oncological reasons were included as well. Finally, in the last column, there is the number of patients prepared in the Surgery Admission Unit during the same period in 2008. The mean number of daily preparations using the chairs was 15, ranging from 12 to 24. The percentage of patients that could not be admitted to a chair was 5% (IC 95%: 3.7%-6.3).

**Table 1:** Surgery procedures and Surgery Admission Unit activity.

	2006		2008		
		
Speciality	n	n (%) oncologic admissions	n	n (%) oncologic admissions	Surgery admission unit (SAU) (%)
Vascular surgery	165	-	137	1 (1)	13 (9)
Cardiac surgery	254	-	210	-	-
General surgery	582	137 (24)	557	162 (29)	167 (30)
Maxilophacial	56	17 (30)	66	19 (29)	29 (44)
Plastic surgery	193	95 (49)	228	117 (51)	118 (52)
Thoracic surgery	127	49 (39)	121	49 (40)	97 (80)
Gynaecological	208	118 (57)	192	118 (61)	21 (11)
Neurosurgery	152	31 (20)	187	38 (20)	66 (35)
Ophthalmology	68	4 (6)	117	14 (12)	36 (31)
Otorrinolaringology	332	64 (19)	320	68 (21)	112 (35)
Traumatology and orthopedics	433	8 (2)	445	5 (1)	212 (48)
Urology	433	208 (48)	470	198 (42)	160 (34)

Total	3003	731 (24)	3050	789(26)	1031 (34)

From the list of theatre incidences, we found that in 2006 there were 91 patients that were delayed because of late preparation and, in 2008, the number of these patients was 81(2.7%, IC 95%: 2.5-2.9) and 22 (2.2%, IC 95%: 2.1-2.3) of them were coming from the Surgery Admission Unit. Another reason for delays was not having the clinical files available with the patient. For this reason the number of delayed interventions was 59 in 2006 and 36 in 2008. The number of cancelled interventions due to insufficient preparation was 31 patients in 2006 and 7 patients in 2008.

The proportion of patients admitted on the same day as surgery was 67% (IC 95%:65%-69%) in 2006 and 76% (IC 95%:74%-78%) in 2008 (p < 0.005).

The patients' global length of stay included in our study was 6.2 days (IC 95%:6-6.4) in 2006 and 5.5 days (IC 95%:5.2-5.8) in 2008 (p < 0.005). Pre-surgery length of stay was reduced from 0.46 days (IC 95%:0.44-0.48) in 2006 to 0.29 days (IC 95%:0.27-0.31) in 2008 (p < 0.005).

The mean number of emergency patients without a bed at 8:00 am was 4.7 (IC 95%:4.29-5.11) patients per day in 2006 and 3.3 (IC 95%:2.93-3.67) patients per day in 2008 (P < 0.005). The percentage of emergency visits that were finally admitted to the hospital was 10.1% (IC 95%:7%-13%) in 2006 and 10.6% (IC 95%:7%-14%) in 2008. The percentage of emergency admissions over global admissions was 50.5% (IC 95%:45%-56%) in 2006 and 49.0% (IC 95%:44%-54%) in 2008.

## Discussion

The implementation of a Surgery Admission Unit for patients undergoing elective surgery has proved to be an effective strategy to shorten the length of stay and increase the proportion of patients admitted on the same day as surgery. In addition, the number of beds unnecessarily occupied for elective admissions decreased and therefore there were more beds available for emergency admissions. In consequence, with the same admissions there should have been less emergency patients waiting for a bed in the following morning at the ED. As we have seen in this study, the admission process and, therefore, variations in length of stay are largely in our control. There is a significant opportunity to redesign the patients' pathways and create important benefits for bed management. In our study, the surgery admission unit achieved a better bed usage allowing a mean number of 14 available beds daily, which may have prevented surgery cancellations or emergency admissions kept waiting for a bed.

The way hospital beds are managed affects the way other departments--such as operating theatres and ED-- perform since they are dependent on bed availability [13]. In turn, these other hospital departments have an impact on bed usage [14,15]. Departments that are inefficient can lengthen hospital stays and use beds unnecessarily [16]. Bed management issues therefore warrant high consideration within the hospital's management team. Some management teams have recognised this and the person in charge of patient pathways management is a member of the hospital's executive committee.

Almost 50 percent of hospital admissions involve non-emergency patients who have been on a waiting list, mostly for a surgical operation [3]. Waiting dominates many citizens' impressions of hospital care. While they are waiting, patients may be in considerable pain and discomfort and this interferes with their normal lifestyle and it adds to the workload of primary care. In this context, elective surgery admissions in our hospital were traditionally admitted the day before in order to avoid cancellations because of a lack of beds. As a result, some beds were used unnecessarily [17] and that had a strong impact on emergency admissions. Some changes have been performed in order to gain efficiency in bed management, such as considering day surgery as the first option for some surgery processes [18], start planning discharge from the admission point through the short stay surgery units, which included patients that underwent surgery or diagnostic processes who had an expected length of stay under 72 hours and, finally, to change the admission process for elective surgery by promoting admission on the same day.

However, the surgery admission unit had some drawbacks. The most important was the delay with bed assignment when the surgery was finished. In this context, when patients were admitted each morning there were not any free beds in the hospital wards, and they had to wait until other patients left the hospital. In consequence, this delay impacted the rotation of patients in recovery theatres after the surgery and, finally, in operating theatres. At that particular time we did not have data to support this statement but improvements in hospital information systems are going to provide this information in the future. Another limitation of this unit was that some patients could not be admitted to a chair, so when planning patient admission and chair allocation, the admission staff had to take into account special needs and exceptions to this process. In addition, this intervention was implemented in only one hospital, so the study's generalizability is limited. We did not implement any other initiative in our hospital during the same period that could account for the differences observed.

In our experience, it is crucial that when the leaders of the hospital management team focus on efforts to promote admission on the same day as surgery, they should also promote early hospital discharge so that the patients admitted without a bed can be placed in the most appropriate bed as soon as possible after the surgery. In planned admissions, the discharge process should start at the point of admission as it is the mismatch between demand and capacity that creates the queues and bottlenecks in the system.

The most important limitation of this study was that we did not examine the cost-effectiveness of this intervention. However, the provision of beds, and all the supplies involved, accounts for much of the health systems' most expensive activity. According to this study we have shortened the length of stay of surgery patients and we can affirm that there were bed savings for the same health service provision. In addition, the rotation index per day of the chairs from the Surgery Admission Unit was at least three patients. All these results lead to the conclusion that this intervention could be cost effective.

## Conclusion

The efficient and effective management of inpatient beds is essential for the benefit of both patients and the hospital. This means that patients are admitted promptly to an appropriate bed and stay for no longer than is necessary. With the results that were reached with the surgery admission unit we have assessed a significant improvement in bed utilisation efficiency. Due to the shorter length of stay that was achieved we can say that our results could be associated with a decrease in overall costs to the hospital.

## Competing interests

The authors declare that they have no competing interests.

## Authors' contributions

BO contributed to conception and design, acquisition of data, performed the statistical analysis and interpretation of data, as well as drafting the manuscript and adding all the comments from other authors; CC contributed to conception of the study as well as to the interpretation of data and to drafting the discussion of the manuscript; SA contributed to revising the manuscript critically for important intellectual content; VMF participated in participated in the program design and coordination and in the acquisition of data and in revising the draft manuscript; BC participated in revising the manuscript critically for important intellectual content; CX participated in revising the manuscript critically for important intellectual content.

All authors read and approved the final manuscript.

## Pre-publication history

The pre-publication history for this paper can be accessed here:

http://www.biomedcentral.com/1472-6963/10/23/prepub
